# 2,3-Dibromo-3-phenyl­propionic acid

**DOI:** 10.1107/S160053680802919X

**Published:** 2008-09-17

**Authors:** Pui Yee Thong, Kong Mun Lo, Seik Weng Ng

**Affiliations:** a Department of Chemistry, University of Malaya, 50603 Kuala Lumpur, Malaysia

## Abstract

In the crystal of the title compound, C_9_H_8_Br_2_O_2_, inversion dimers linked by two O—H⋯O hydrogen bonds occur. All of the carbon and oxygen atoms are disordered over two sets of sites in a 2:1 ratio.

## Related literature

For *threo*-1,2-diphenyl-2,3-difluoro­propionate, see: O’Hagan *et al.* (2006 ▶). For *R*-methyl 3-bromo-2-chloro-3-phenyl­propionate, see: Shaw *et al.* (1995 ▶).
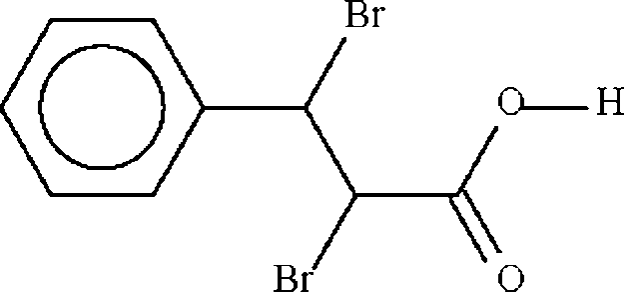


## Experimental

### 

#### Crystal data

C_9_H_8_Br_2_O_2_
*M* *_r_* = 307.97
Orthorhombic, 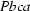

*a* = 7.0278 (1) Å
*b* = 9.7105 (1) Å
*c* = 29.2970 (4) Å
*V* = 1999.33 (4) Å^3^
*Z* = 8
Mo *K*α radiation
μ = 8.07 mm^−1^
*T* = 100 (2) K
0.28 × 0.22 × 0.14 mm

#### Data collection

Bruker SMART APEX diffractometer
Absorption correction: multi-scan (*SADABS*; Sheldrick, 1996 ▶) *T*_min_ = 0.211, *T*_max_ = 0.398 (expected range = 0.171–0.323)
17420 measured reflections
2303 independent reflections
2056 reflections with *I* > 2σ(*I*)
*R*_int_ = 0.027

#### Refinement

*R*[*F* ^2^ > 2σ(*F* ^2^)] = 0.025
*wR*(*F* ^2^) = 0.066
*S* = 1.06
2303 reflections
127 parameters
83 restraints
H-atom parameters constrained
Δρ_max_ = 0.61 e Å^−3^
Δρ_min_ = −0.62 e Å^−3^

### 

Data collection: *APEX2* (Bruker, 2007 ▶); cell refinement: *SAINT* (Bruker, 2007 ▶); data reduction: *SAINT*; program(s) used to solve structure: *SHELXS97* (Sheldrick, 2008 ▶); program(s) used to refine structure: *SHELXL97* (Sheldrick, 2008 ▶); molecular graphics: *X-SEED* (Barbour, 2001 ▶); software used to prepare material for publication: *publCIF* (Westrip, 2008 ▶).

## Supplementary Material

Crystal structure: contains datablocks global, I. DOI: 10.1107/S160053680802919X/tk2304sup1.cif

Structure factors: contains datablocks I. DOI: 10.1107/S160053680802919X/tk2304Isup2.hkl

Additional supplementary materials:  crystallographic information; 3D view; checkCIF report

## Figures and Tables

**Table 1:** Hydrogen-bond geometry (Å, °)

*D*—H⋯*A*	*D*—H	H⋯*A*	*D*⋯*A*	*D*—H⋯*A*
O1—H1o⋯O2^i^	0.84	1.86	2.68 (1)	165
O1′—H1′o⋯O2^i^	0.84	1.86	2.69 (1)	166

Symmetry code: (i) 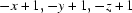.

**Fig. 1.**
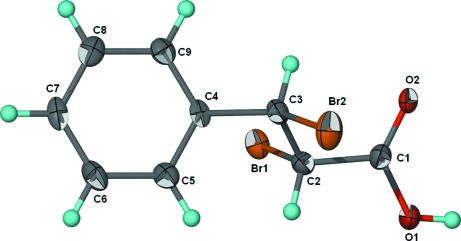
70% Probability thermal ellipsoid plot (Barbour, 2001) of C9H8Br2O2. Hydrogen atoms are drawn as spheres of arbitrary radii. For clarity, the minor component is not shown.

## supplementary crystallographic information

### Comment

The compound (Scheme I, Fig. 1) was obtained as a side-product from the reaction of cyclopentyldiphenyltin cinnamate and 4-dimethylaminopyridine hydrobromide perbromide. Possibly, bromine added across the double bond of the cinnamate group followed by cleavage of the tin–carbon bond. Only few dihalogenproponic acid derivatives have been characterized by X-ray crystallography. These are limited to, for example, *threo*-methyl 2,3-difluoropropionate (O'Hagan *et al.*, 2006) and *R*-methyl 3-bromo-2-chloro-3-phenylpropionate (Shaw *et al.*, 1995).

### Experimental

The compound was obtained as a side-product from the reaction of cyclopentyldiphenyltin cinnamate (0.3 g, 0.6 mmol) and 4-dimethylaminopyridine hydrobromide perbromide (0.25 g) in a mixture of chloroform and ethanol.

### Refinement

The structure is disordered over two positions with respect of the non-bromide atoms. The Br1 atom is connected to the carbon atom in the 2-position in the major component but is connected to the carbon atom in the 3-position in the minor component. Conversely, the Br2 atom is connected to the carbon atom in the 3-position in the major component but is connected to the carbon atom in the 2-position in the minor component. All distances in the major component were restrained to within 0.01 Å of their equivalents in the minor component. The phenyl rings were restrained into rigid hexagons of 1.39 Å sides. Additionally, the four-atom carboxyl and seven-atom benzyl units were each restrained to be nearly flat. The anisotropic displacement parameters of the primed atoms were restrained to be equal to those of the unprimed atoms; these were also restrained to be nearly isotropic. In the inital stages of the refinement, the occupancy refined to an approximate 2:1 ratio. However, with the inclusion of hydrogen atoms, the refinement was unstable. Ratios that were either slightly smaller or slightly larger than 2:1 did not yield any significant differences in the final residual index. The ratio was then fixed to 2:1 Oxygen and carbon-bound H-atoms were placed in calculated positions (C—H 0.95 to 1.00 Å, O–H 0.84 Å) and were included in the refinement in the riding model approximation, with *U*(H) set to 1.2 to 1.5*U*_eq_(C,*O*).

### Crystal data

**Table:**

C_9_H_8_Br_2_O_2_	*F*(000) = 1184
*M**_r_* = 307.97	*D*_x_ = 2.046 Mg m^−^^3^
Orthorhombic, *P**b**c**a*	Mo *K*α radiation, λ = 0.71073 Å
Hall symbol: -P 2ac 2ab	Cell parameters from 8127 reflections
*a* = 7.0278 (1) Å	θ = 2.2–28.2°
*b* = 9.7105 (1) Å	µ = 8.07 mm^−^^1^
*c* = 29.2970 (4) Å	*T* = 100 K
*V* = 1999.33 (4) Å^3^	Block, colorless
*Z* = 8	0.28 × 0.22 × 0.14 mm

### Data collection

**Table:**

Bruker SMART APEX diffractometer	2303 independent reflections
Radiation source: fine-focus sealed tube	2056 reflections with *I* > 2σ(*I*)
graphite	*R*_int_ = 0.027
ω scans	θ_max_ = 27.5°, θ_min_ = 2.8°
Absorption correction: multi-scan (SADABS; Sheldrick, 1996)	*h* = −9→9
*T*_min_ = 0.211, *T*_max_ = 0.398	*k* = −12→12
17420 measured reflections	*l* = −37→38

### Refinement

**Table:**

Refinement on *F*^2^	Primary atom site location: structure-invariant direct methods
Least-squares matrix: full	Secondary atom site location: difference Fourier map
*R*[*F*^2^ > 2σ(*F*^2^)] = 0.025	Hydrogen site location: inferred from neighbouring sites
*wR*(*F*^2^) = 0.066	H-atom parameters constrained
*S* = 1.06	*w* = 1/[σ^2^(*F*_o_^2^) + (0.0337*P*)^2^ + 3.2334*P*] where *P* = (*F*_o_^2^ + 2*F*_c_^2^)/3
2303 reflections	(Δ/σ)_max_ = 0.001
127 parameters	Δρ_max_ = 0.61 e Å^−^^3^
83 restraints	Δρ_min_ = −0.62 e Å^−^^3^

### Fractional atomic coordinates and isotropic or equivalent isotropic displacement parameters (Å^2^)

**Table:**

	*x*	*y*	*z*	*U*_iso_*/*U*_eq_	Occ. (<1)
Br1	0.48392 (4)	0.31033 (3)	0.364350 (9)	0.02208 (9)	
Br2	−0.06355 (4)	0.45596 (3)	0.436758 (9)	0.03094 (10)	
O1	0.4216 (6)	0.3279 (4)	0.48256 (13)	0.0308 (10)	0.67
H1O	0.4765	0.3569	0.5061	0.046*	0.67
O2	0.4214 (10)	0.5338 (5)	0.4476 (3)	0.0209 (10)	0.67
C1	0.3847 (4)	0.4118 (4)	0.44922 (12)	0.0189 (8)	0.67
C2	0.2859 (5)	0.3388 (4)	0.40959 (12)	0.0176 (7)	0.67
H2	0.2344	0.2480	0.4200	0.021*	0.67
C3	0.1313 (5)	0.4223 (4)	0.38839 (12)	0.0183 (7)	0.67
H3	0.1859	0.5130	0.3790	0.022*	0.67
C4	0.0358 (4)	0.3575 (5)	0.34721 (14)	0.0133 (8)	0.67
C5	−0.0307 (8)	0.2228 (5)	0.3486 (3)	0.0186 (11)	0.67
H5	−0.0167	0.1697	0.3756	0.022*	0.67
C6	−0.1176 (9)	0.1657 (9)	0.3104 (4)	0.0224 (6)	0.67
H6	−0.1631	0.0736	0.3114	0.027*	0.67
C7	−0.1381 (9)	0.2433 (15)	0.2709 (3)	0.0181 (8)	0.67
H7	−0.1976	0.2043	0.2448	0.022*	0.67
C8	−0.0716 (13)	0.3781 (14)	0.26947 (16)	0.0206 (7)	0.67
H8	−0.0856	0.4311	0.2424	0.025*	0.67
C9	0.0154 (11)	0.4352 (8)	0.3076 (2)	0.0183 (9)	0.67
H9	0.0608	0.5273	0.3067	0.022*	0.67
O1'	0.3463 (14)	0.3474 (10)	0.4912 (3)	0.0308 (10)	0.33
H1'O	0.4202	0.3715	0.5122	0.046*	0.33
O2'	0.380 (2)	0.5445 (11)	0.4542 (7)	0.0209 (10)	0.33
C1'	0.3118 (9)	0.4305 (9)	0.4576 (2)	0.0189 (8)	0.33
C2'	0.1737 (9)	0.3684 (7)	0.4228 (2)	0.0176 (7)	0.33
H2'	0.1633	0.2666	0.4274	0.021*	0.33
C3'	0.2239 (9)	0.3993 (8)	0.3748 (2)	0.0183 (7)	0.33
H3'	0.2355	0.5012	0.3709	0.022*	0.33
C4'	0.0862 (9)	0.3432 (12)	0.3394 (3)	0.0133 (8)	0.33
C5'	0.0090 (19)	0.2117 (12)	0.3425 (6)	0.0186 (11)	0.33
H5'	0.0421	0.1536	0.3674	0.022*	0.33
C6'	−0.1164 (19)	0.165 (2)	0.3092 (8)	0.0224 (6)	0.33
H6'	−0.1692	0.0754	0.3113	0.027*	0.33
C7'	−0.1648 (18)	0.250 (3)	0.2729 (6)	0.0181 (8)	0.33
H7'	−0.2505	0.2185	0.2501	0.022*	0.33
C8'	−0.088 (3)	0.382 (3)	0.2698 (4)	0.0206 (7)	0.33
H8'	−0.1207	0.4398	0.2449	0.025*	0.33
C9'	0.038 (2)	0.4282 (17)	0.3031 (5)	0.0183 (9)	0.33
H9'	0.0906	0.5181	0.3010	0.022*	0.33

### Atomic displacement parameters (Å^2^)

**Table:**

	*U*^11^	*U*^22^	*U*^33^	*U*^12^	*U*^13^	*U*^23^
Br1	0.02081 (14)	0.02271 (15)	0.02272 (15)	−0.00251 (10)	−0.00187 (10)	−0.00479 (10)
Br2	0.03118 (17)	0.04015 (19)	0.02150 (15)	0.00134 (13)	0.00331 (11)	−0.00921 (12)
O1	0.055 (3)	0.0188 (16)	0.0182 (18)	−0.0063 (19)	−0.0143 (18)	0.0006 (13)
O2	0.034 (3)	0.0169 (12)	0.012 (3)	0.0006 (16)	−0.0033 (18)	−0.0032 (12)
C1	0.023 (2)	0.0199 (18)	0.0139 (17)	−0.0030 (19)	−0.0010 (16)	−0.0003 (14)
C2	0.0211 (17)	0.0149 (15)	0.0166 (16)	−0.0017 (14)	0.0007 (13)	−0.0010 (12)
C3	0.021 (2)	0.0159 (16)	0.0175 (17)	−0.0033 (15)	0.0002 (14)	−0.0028 (13)
C4	0.007 (2)	0.0153 (17)	0.017 (2)	0.0062 (19)	0.0015 (16)	−0.0077 (14)
C5	0.016 (3)	0.0174 (16)	0.023 (3)	0.0023 (17)	−0.002 (2)	−0.0009 (15)
C6	0.0180 (12)	0.0183 (13)	0.0310 (15)	−0.0018 (10)	−0.0052 (11)	−0.0067 (11)
C7	0.009 (2)	0.0239 (19)	0.0215 (14)	0.006 (2)	−0.0035 (16)	−0.0106 (11)
C8	0.0169 (19)	0.0259 (15)	0.0190 (12)	0.0082 (15)	0.0007 (11)	−0.0025 (10)
C9	0.017 (2)	0.0167 (14)	0.0211 (18)	0.0027 (13)	0.0015 (14)	−0.0037 (14)
O1'	0.055 (3)	0.0188 (16)	0.0182 (18)	−0.0063 (19)	−0.0143 (18)	0.0006 (13)
O2'	0.034 (3)	0.0169 (12)	0.012 (3)	0.0006 (16)	−0.0033 (18)	−0.0032 (12)
C1'	0.023 (2)	0.0199 (18)	0.0139 (17)	−0.0030 (19)	−0.0010 (16)	−0.0003 (14)
C2'	0.0211 (17)	0.0149 (15)	0.0166 (16)	−0.0017 (14)	0.0007 (13)	−0.0010 (12)
C3'	0.021 (2)	0.0159 (16)	0.0175 (17)	−0.0033 (15)	0.0002 (14)	−0.0028 (13)
C4'	0.007 (2)	0.0153 (17)	0.017 (2)	0.0062 (19)	0.0015 (16)	−0.0077 (14)
C5'	0.016 (3)	0.0174 (16)	0.023 (3)	0.0023 (17)	−0.002 (2)	−0.0009 (15)
C6'	0.0180 (12)	0.0183 (13)	0.0310 (15)	−0.0018 (10)	−0.0052 (11)	−0.0067 (11)
C7'	0.009 (2)	0.0239 (19)	0.0215 (14)	0.006 (2)	−0.0035 (16)	−0.0106 (11)
C8'	0.0169 (19)	0.0259 (15)	0.0190 (12)	0.0082 (15)	0.0007 (11)	−0.0025 (10)
C9'	0.017 (2)	0.0167 (14)	0.0211 (18)	0.0027 (13)	0.0015 (14)	−0.0037 (14)

### Geometric parameters (Å, °)

**Table:**

Br1—C2	1.942 (4)	C8—H8	0.9500
Br1—C3'	2.044 (6)	C9—H9	0.9500
Br2—C2'	1.916 (6)	O1'—C1'	1.295 (8)
Br2—C3	1.997 (3)	O1'—H1'O	0.8400
O1—C1	1.298 (4)	O2'—C1'	1.212 (8)
O1—H1O	0.8400	C1'—C2'	1.533 (8)
O2—C1	1.213 (5)	C2'—C3'	1.480 (7)
C1—C2	1.527 (5)	C2'—H2'	1.0000
C2—C3	1.491 (5)	C3'—C4'	1.518 (7)
C2—H2	1.0000	C3'—H3'	1.0000
C3—C4	1.517 (4)	C4'—C5'	1.3900
C3—H3	1.0000	C4'—C9'	1.3900
C4—C5	1.3900	C5'—C6'	1.3900
C4—C9	1.3900	C5'—H5'	0.9500
C5—C6	1.3900	C6'—C7'	1.3900
C5—H5	0.9500	C6'—H6'	0.9500
C6—C7	1.3900	C7'—C8'	1.3900
C6—H6	0.9500	C7'—H7'	0.9500
C7—C8	1.3900	C8'—C9'	1.3900
C7—H7	0.9500	C8'—H8'	0.9500
C8—C9	1.3900	C9'—H9'	0.9500
			
C1—O1—H1O	120.0	C1'—O1'—H1'O	120.0
O2—C1—O1	126.8 (6)	O2'—C1'—O1'	124.0 (13)
O2—C1—C2	121.4 (6)	O2'—C1'—C2'	123.8 (13)
O1—C1—C2	111.8 (4)	O1'—C1'—C2'	112.3 (8)
C3—C2—C1	113.3 (3)	C3'—C2'—C1'	113.7 (6)
C3—C2—Br1	108.4 (2)	C3'—C2'—Br2	108.7 (4)
C1—C2—Br1	105.0 (2)	C1'—C2'—Br2	103.5 (4)
C3—C2—H2	110.0	C3'—C2'—H2'	110.2
C1—C2—H2	110.0	C1'—C2'—H2'	110.2
Br1—C2—H2	110.0	Br2—C2'—H2'	110.2
C2—C3—C4	115.4 (3)	C2'—C3'—C4'	115.0 (6)
C2—C3—Br2	107.0 (2)	C2'—C3'—Br1	105.6 (4)
C4—C3—Br2	109.20 (19)	C4'—C3'—Br1	108.5 (4)
C2—C3—H3	108.3	C2'—C3'—H3'	109.2
C4—C3—H3	108.3	C4'—C3'—H3'	109.2
Br2—C3—H3	108.3	Br1—C3'—H3'	109.2
C5—C4—C9	120.0	C5'—C4'—C9'	120.0
C5—C4—C3	121.0 (5)	C5'—C4'—C3'	122.3 (11)
C9—C4—C3	119.0 (5)	C9'—C4'—C3'	117.7 (11)
C4—C5—C6	120.0	C4'—C5'—C6'	120.0
C4—C5—H5	120.0	C4'—C5'—H5'	120.0
C6—C5—H5	120.0	C6'—C5'—H5'	120.0
C7—C6—C5	120.0	C7'—C6'—C5'	120.0
C7—C6—H6	120.0	C7'—C6'—H6'	120.0
C5—C6—H6	120.0	C5'—C6'—H6'	120.0
C6—C7—C8	120.0	C6'—C7'—C8'	120.0
C6—C7—H7	120.0	C6'—C7'—H7'	120.0
C8—C7—H7	120.0	C8'—C7'—H7'	120.0
C7—C8—C9	120.0	C7'—C8'—C9'	120.0
C7—C8—H8	120.0	C7'—C8'—H8'	120.0
C9—C8—H8	120.0	C9'—C8'—H8'	120.0
C8—C9—C4	120.0	C8'—C9'—C4'	120.0
C8—C9—H9	120.0	C8'—C9'—H9'	120.0
C4—C9—H9	120.0	C4'—C9'—H9'	120.0
			
O2—C1—C2—C3	−40.3 (4)	O2'—C1'—C2'—Br2	−77.8 (5)
O1—C1—C2—C3	140.0 (3)	O1'—C1'—C2'—Br2	102.1 (5)
O2—C1—C2—Br1	77.8 (3)	C3—Br2—C2'—C3'	0.9 (4)
O1—C1—C2—Br1	−101.9 (2)	C3—Br2—C2'—C1'	122.1 (7)
C1—C2—C3—C4	176.8 (3)	C1'—C2'—C3'—C4'	−178.0 (6)
Br1—C2—C3—C4	60.7 (4)	Br2—C2'—C3'—C4'	−63.3 (7)
C1—C2—C3—Br2	−61.5 (3)	C1'—C2'—C3'—Br1	62.4 (6)
Br1—C2—C3—Br2	−177.59 (16)	Br2—C2'—C3'—Br1	177.1 (3)
C2—C3—C4—C5	49.8 (4)	C2—Br1—C3'—C2'	0.1 (3)
Br2—C3—C4—C5	−70.8 (3)	C2—Br1—C3'—C4'	−123.7 (8)
C2—C3—C4—C9	−130.2 (4)	C2'—C3'—C4'—C5'	−42.3 (8)
Br2—C3—C4—C9	109.2 (3)	Br1—C3'—C4'—C5'	75.6 (6)
C9—C4—C5—C6	0.0	C2'—C3'—C4'—C9'	137.7 (8)
C3—C4—C5—C6	−179.99 (8)	Br1—C3'—C4'—C9'	−104.3 (6)
C4—C5—C6—C7	0.0	C9'—C4'—C5'—C6'	0.0
C5—C6—C7—C8	0.0	C3'—C4'—C5'—C6'	−179.96 (9)
C6—C7—C8—C9	0.0	C4'—C5'—C6'—C7'	0.0
C7—C8—C9—C4	0.0	C5'—C6'—C7'—C8'	0.0
C5—C4—C9—C8	0.0	C6'—C7'—C8'—C9'	0.0
C3—C4—C9—C8	179.99 (8)	C7'—C8'—C9'—C4'	0.0
O2'—C1'—C2'—C3'	40.0 (6)	C5'—C4'—C9'—C8'	0.0
O1'—C1'—C2'—C3'	−140.1 (6)	C3'—C4'—C9'—C8'	179.96 (8)

### Hydrogen-bond geometry (Å, °)

**Table:**

*D*—H···*A*	*D*—H	H···*A*	*D*···*A*	*D*—H···*A*
O1—H1o···O2^i^	0.84	1.86	2.68 (1)	165
O1'—H1'o···O2^i^	0.84	1.86	2.69 (1)	166

Symmetry codes: (i) −*x*+1, −*y*+1, −*z*+1.
